# Adverse Effect of Metallic Gold and Silver Nanoparticles on *Xenopus laevis* Embryogenesis

**DOI:** 10.3390/nano13172488

**Published:** 2023-09-04

**Authors:** Rosa Carotenuto, Margherita Tussellino, Sabato Fusco, Giovanna Benvenuto, Fabio Formiggini, Bice Avallone, Chiara Maria Motta, Chiara Fogliano, Paolo Antonio Netti

**Affiliations:** 1 Department of Biology, University of Naples Federico II, 80126 Naples, Italy; 2 Department of Medicine and Health Sciences “Vincenzo Tiberio”, University of Molise, 86100 Campobasso, Italy; 3 Stazione Zoologica “Anton Dohrn”, 80122 Naples, Italy; 4 Center for Advanced Biomaterials for Health Care (IIT@CRIB), Italian Institute of Technology, 80125 Naples, Italy; 5 Department of Chemical Materials and Industrial Production (DICMAPI), University of Naples Federico II, 80125 Naples, Italy

**Keywords:** FETAX test, nanoparticles, transmission electron microscopy, gene expression

## Abstract

Exposure to metal nanoparticles is potentially harmful, particularly when occurring during embryogenesis. In this study, we tested the effects of commercial AuNPs and AgNPs, widely used in many fields for their features, on the early development of *Xenopus laevis*, an anuran amphibian key model species in toxicity testing. Through the Frog Embryo Teratogenesis Assay—*Xenopus* test (FETAX), we ascertained that both nanoparticles did not influence the survival rate but induced morphological anomalies like modifications of head and branchial arch cartilages, depigmentation of the dorsal area, damage to the intestinal brush border, and heart rate alteration. The expression of genes involved in the early pathways of embryo development was also modified. This study suggests that both types of nanoparticles are toxic though nonlethal, thus indicating that their use requires attention and further study to better clarify their activity in animals and, more importantly, in humans.

## 1. Introduction

In recent years, the scientific community has shown particular interest in nanotechnology, which represents a radically new way of producing materials, structures, and devices with significantly improved or entirely new properties and functionalities. Manipulation at atomic and molecular scales allows for the creation of new materials, made up of particles between 1 and 100 nm in size, referred to as nanoparticles (NPs), as indicated by the American Society for Testing and Materials (ASTM) [1]. The revolutionary perspectives associated with nanotechnology derive from the fact that, at these reduced dimensions, the behavior and characteristics of materials change drastically compared to larger dimensions. Because of this, their applications in the different productive sectors are potentially infinite. Based on these premises, the problem of the associated risk arises. Nanoparticles are nonbiodegradable and can remain in organisms, depositing in various organs. The very same innovative chemical–physical characteristics that make NPs advantageous for applications (surface area, surface reactivity, state of aggregation/agglomeration, composition, surface charge, solubility, shape, and porosity) lead to unique and entirely new biological effects but they are also a cause of toxicity [2,3,4,5]. Despite SCHENIR, FDA, and WHO directives, it is still unclear how to assess their toxicity, and toxicological and non-toxicological assays are underway to understand their properties and quantify the relationship between possible risks and benefits from their intake [5]. Given the wide range of applications, NPs of different sizes and shapes are needed; therefore, a variety of protocols are necessary to produce monodisperse nanoparticles with controlled morphology. The development of metallic nanoparticles is very rapid and multidirectional and the main chemical method for their synthesis is reduction, but green synthesis is also applied [5]. Among the different types of metal nanomaterials, gold (AuNPs) and silver (AgNPs) nanoparticles have attracted great interest from different fields of science due to their particular features, which make them versatile for use in different areas such as engineering, medicine, chemistry, and physics [5,6,7,8]. Among the others, AuNPs are employed as extraordinary scaffolds for various applications in therapeutics, detection and diagnostics, chemical and biological sensing, biolabeling, and sensing but have also been used for drug delivery and delivery into cells [6,7,8,9,10]. Although composed of an inert material, gold nanoparticles can adsorb molecules on the surface and easily penetrate the whole conjugate, and once internalized, are stored in cells’ vesicular structures close to the cell nucleus [11]. The long-term implications of this internalization remain to be clarified. Silver nanoparticles have also attracted remarkable attention due to their powerful antimicrobial effects. For this reason, they have found vast applications in medical devices, fabrics, cosmetics, personal hygiene products, toys, plastics, and electronics [12,13]. If engineered down to the nano dimension, the antibacterial properties are amplified, so much so that they are used as an antibacterial and antifungal agent in biotechnology and bioengineering, textile engineering, and water treatment [13,14]. Moreover, AgNPs have antiviral properties since they induce the production of free radicals and reactive oxygen species (ROS; 7), which is why their use markedly increased during the recent COVID-19 pandemic [15,16]. As a consequence of their large-scale use, AuNPs and AgNPs are among the largest groups of manufactured NPs present in the environment, which has led to their accumulation in food webs [3,17,18]. The aquatic environment in particular is at risk of exposure since water is the preferred spill site for most wastes. Unfortunately, little is known about the impact of exposure in aquatic non-target organisms and the relationship existing between exposure scenarios and toxicological consequences. Whilst data indicate that nanoparticles, in general, are potentially harmful to aquatic organisms [4,19,20], sufficient evidence is lacking for AuNPs and AgNPs, whose potential on aquatic biota is still unclear, especially for amphibians.

Therefore, this paper aims to assess the toxic potential of these two commonly used nanoparticles on the embryonic development of the anuran amphibian *Xenopus laevis*, an excellent model system for toxicity testing and a valid model for assessing nanoparticles’ harmfulness [4,21]. Studies were conducted using a modified FETAX assay, developed specifically for the application of toxicity testing in this organism and to simulate environmental exposure [4,22]. Gold and silver nanoparticles, with a manufacturer-claimed diameter of 20 nm, were first characterized with dynamic light scattering (DLS) and size and morphology were checked with transmission electron microscopy. Their toxic effect was assessed on embryonic development evaluating mortality, rate of phenotypic alterations, and induction of ROS production. In addition, since the literature data indicate that NPs can modify gene expression [23] we investigated whether AuNPs and AgNPs alter the expression of some master genes in early embryonic development. In particular, *bmp4*, *fgf8*, *pax6*, *rax1*, *egr2*, and *sox9* were chosen since they are involved in axes formation, gastrulation, and neurulation and are markers of different encephalic regions and neural crest migration pathways [4,22,23,24,25].

## 2. Materials and Methods

### 2.1. Animals

Adult *Xenopus laevis*, obtained from Nasco (Fort Atkinsons, WI, USA), were stabled at the Department of Biology of the University of Naples “Federico II”, according to the guidelines of the University Animal Welfare Office and the recommendations of the Guide for the Care and Use of Laboratory Animals of the National Institutes of Health of the Italian Ministry of Health (authorization DL 116/92). The protocol was approved by the Committee on the Ethics of Animal Experiments (Centro Servizi Veterinari) of the University of Naples Federico II (Permit Number: 05/2016-UT; D.lgs 26/2014). To perform in vitro fertilization, the eggs were obtained by injecting females in the dorsal lymphatic sac with 500 units of Gonase (AMSA) dissolved in amphibian Ringer solution (111 mM NaCl, 1.3 mM CaCl_2_, 2 mM KCl, 0.8 mM MgSO_4_, in 25 mM Hepes, pH 7.4). Fertilized eggs and embryos were obtained using standard insemination methods, as reported by Carotenuto et al. [26] and staged according to Nieuwkoop and Faber [27].

### 2.2. AuNP and AgNP Characterization

AuNPs (MKN-Au-S020; Gold Nanoparticles in Aqueous Media; 7.0 × 10^11^ particles/mL, corresponding to 5 mg/mL. Code: 7115.9090) and AgNPs (MKN-Ag-S020; Silver Nanoparticles in Aqueous Media; Size 20 ± 3 µm, concentrate 5 mg/mL) of 20 µm in diameter were purchased from MKnano-Canada (Mississauga, ON, Canada). NPs’ sizes are within the range of sizes that easily penetrate cells [28] and nuclei [29]. The effective NP diameters and their size distributions were checked using transmission electron microscopy (TEM). Formvar-coated 200 mesh copper grids were used and the excess of water or FETAX was gently blotted using filter paper. Dried grids were directly inserted into a Zeiss LEO 196 912AB EFTEM (Zeiss, Oberkochen, Germany) operating at 100 kV and images were collected at a magnification of ×50,000 using a dedicated CCD. One hundred NPs of each type were measured, and the median, mean, and standard deviation were calculated. Dynamic light scattering (DLS; Zetasizer Nano-ZS; Malvern Instruments, Malvern, UK) analyses were also conducted to verify the size and measure z-potential; the colloidal stability of nanoparticles that, in suspensions, is strongly pH-dependent due to electrostatic repulsion; and also verify the polydispersity index (PdI) values. Measures were conducted at 21 °C, using AuNPs and AgNPs diluted to 1 mg/L in H_2_O, the medium used by the manufacturer, and in amphibian solution (FETAX: pH 7.4, 106 mM NaCl, 11 mM NaHCO_3_, 4 mM KCl, 1 mM CaCl_2_, 4 mM CaSO_4_, 3 mM MgSO_4_) [30]. All measures were performed in triplicate.

### 2.3. Exposure to AuNPs and AgNPs

Three in vitro fertilizations were carried out and, for each female, the test was replicated 3 times for the control group and each of the compound concentration groups. The FETAX assay was modified by anticipating the contact of the embryos with the NPs at stage 4/8 cells to mimic natural conditions [4,22]. The selected embryos were placed and grown in FETAX solution containing AuNPs or AgNPs at the following concentrations: 0.01 mg/L, 1 mg/L or 5 mg/L (see: [4,19,31,32]). The embryos were collected and dejelled with 0.3% β-mercaptoethanol (pH 9.0) for 1/2 min. Normally cleaved embryos were selected for testing and placed in a 10 cm glass Petri dish, each containing 50 mL of either FETAX (for controls) or NPs solutions (treated) and 15 embryos. All embryos were raised and exposed up to stage 45/46 when survivors were collected. At stage 40/41, *X. laevis* embryos open the mouth [27] and ingestion becomes the main route of NPs intake. For each experimental group, the dead embryos were recorded and removed daily. The experiments were carried out at 21 °C, under a 12 h light:12 h dark photoperiod. The pH (7.4) of the solutions in the Petri dishes containing the embryos was checked daily. No variation in pH was registered.

### 2.4. Phenotype Analysis

Phenotype analysis was carried out with a Leica MZ16F UV stereomicroscope (Leica Microsystems s.r.l., Milan, Italy), integrated with LED Ring Light, equipped with a Leica DFC 300Fx camera and IM50 Image Manager Software. After anesthesia in FETAX medium containing 100 mg/L MS-222 (Sigma, Milan, Italy), a photo of each tadpole was taken in the ventral position to check the morphology of the intestinal area and in the dorsal position to determine length, pigmentation and state of craniofacial cartilages, and branchial basket. Embryo length was determined from the photos with the software Adobe Photoshop CS5 (Adobe Systems Software Ireland Ltd., Dublin, Ireland), calibrated with a ruler photographed at the same magnification as the tadpoles. Measurements were carried out along the plane of bilateral symmetry, from the tip of the head to the tip of the tail. The pigment of the dorsal area, between the region of the olfactory bulbs and the spinal cord, was also quantified via two graphic programs: Adobe Photoshop CS5 (to highlight and calculate the degree of basic dorsal pigmentation and to standardize the collected images) and Image-Pro Plus 6.0 (Media Cybernetics, Inc., Rockville, MD, USA), to determine the percentage of pigment present in the relevant anatomic cut out by analyzing the percentage of image pixels with the same grey level. The tadpoles’ heart rate measurement (expressed in beats per minute) was determined under a dissection microscope (Wild, Heerbrugg) by recording the number of heartbeats for 30 s three times. For each type of analysis, data obtained were normalized by dividing each treated embryo measure by the average value of the control tadpoles [19,33].

### 2.5. Transmission Electron Microscopy Analysis

Control and exposed embryos were randomly selected and, after anesthesia, were fixed in 2.5% glutaraldehyde for 18 h at 4 °C. They were washed three times in 0.1 M sodium cacodylate, postfixed with 1% osmium tetroxide at 4 °C for 1hr, rinsed five times with 0.1 M sodium cacodylate buffer, dehydrated in a graded ethanol series, further substituted with propylene oxide and embedded in Epon 812 (TAAB, TAAB Laboratories Equipment Ltd., Newbury, UK), and polymerized at 60 °C. Resin blocks were sectioned with an Ultracut UCT ultramicrotome (Leica, Wetzlar, Germany). Seventy nanometer ultrathin sections were placed on nickel grids, contrasted with 4% aqueous uranyl acetate, rinsed once with a mix of methanol and bi-distilled water (1:1), twice with bi-distilled water, and observed with a Zeiss LEO 196 912AB EFTEM (Zeiss, Oberkochen, Germany).

### 2.6. Confocal Microscopy

Embryos were fixed at stage 45/46 in 4% formaldehyde at 4 °C, stored in 100% methanol at −20 °C, embedded and frozen in Killik (Bio Optica, Milan, Italy), and sectioned at 60 µm thickness. Heart sections were directly observed and photographed using a Leica SP5 confocal laser scanning microscope. Image acquisition was standardized by maintaining laser power, photomultiplier, pinhole aperture, and confocal scanner settings constant for all experiments. Sections were spaced at 0.7 µm intervals, and at 25 × 0.95 an HCX IRAPO L25 × 0.95 W 0.17 water immersion objective was utilized. For each sample, a total number of 80 optical sections (1.4 µm thick) was analyzed. Images were processed using LAS AF confocal software.

### 2.7. Alcian Blue Staining

Skeletal staining was performed on treated and untreated stage 45/46 embryos, as described by Dong et al. [34]. Briefly, 10 embryos for each group were fixed in 4% paraformaldehyde, rinsed in phosphate-buffered saline/0.1% Tween 20 (PBT), and stained for 6 h in 0.05% Alcian Blue in 80% ethanol/20% acetic acid. After destaining overnight in acidic alcohol, specimens were transferred into 1% KOH/3% H_2_O_2_ for 3 h. The tissue was cleared in 0.05% trypsin in saturated sodium tetraborate for another 2 h. Embryos were washed in PBT. The skin was dissected out, the ethmoidal plate cut along the midline and specimens were flat-mounted on slides. The embryos were observed and photographed with a LeicaMZ16F UV stereomicroscope equipped with a Leica DFC 300Fx camera and IM50 Image Manager Software 4.0.

### 2.8. Real-Time PCR

Total RNA, obtained from 10 randomly selected embryos from each pool, was extracted using the Direct-zol RNA Mini Prep kit (ZymoResearch, Irvine, CA, USA); quality was measured using the spectrophotometric method at a 260/280 ratio. RNA was used for cDNA synthesis with the SuperScript Vilo cDNA synthesis kits (Life Technologies, Waltham, MA, USA). Used primers are listed in Table 1. Real-time PCR was performed using Power SYBR Green Master Mix kits (Life Technologies) using the 96-well optical reaction plate in 20 μL total reaction volume. Reactions were conducted on an AriaMx Real-time PCR System. For transcript relative quantification, samples were normalized to ornithine decarboxylase (odc), a housekeeping control to account for possible differences in the quantity and quality of the cDNAs used in the experiments. The magnitude of change in gene expression relative to control was determined by the 2 −ΔΔCt method of Livak and Schmittgen [35].

### 2.9. Oxygen Reactive Species Analysis

To quantify the production of total oxygen reactive species, proteins of total extracts of embryos were quantized using the BCA method (Pierce™ BCA Protein Assay Kit) and subjected to the test of lipid peroxidation. Embryos pools from controls and NPs-treated were homogenized in 50 mM phosphate buffer pH 7.5, then centrifuged for 30 min at 13,000 rpm, at 4 °C with Eppendorf MiniSpin (Eppendorf™ 5453000011). From each sample, 550 µL of supernatant were taken, and an equal volume of 5% TCA (trichloroacetic acid) and 1% TBA (thiobarbituric acid) were added. After centrifugation at 3000 rpm for 10 min at room temperature, the resulting supernatants were taken, boiled for 10 min and let cool before reading with the spectrophotometer (U-2900, Hitachi, Tokyo, Japan) at a wavelength of 535 nm [36]. The amount of TBARS was expressed as nanomoles of TBARS per milligram of protein.

## 3. Results

### 3.1. AuNPs and AgNPs Characterization

**Figure 1:**
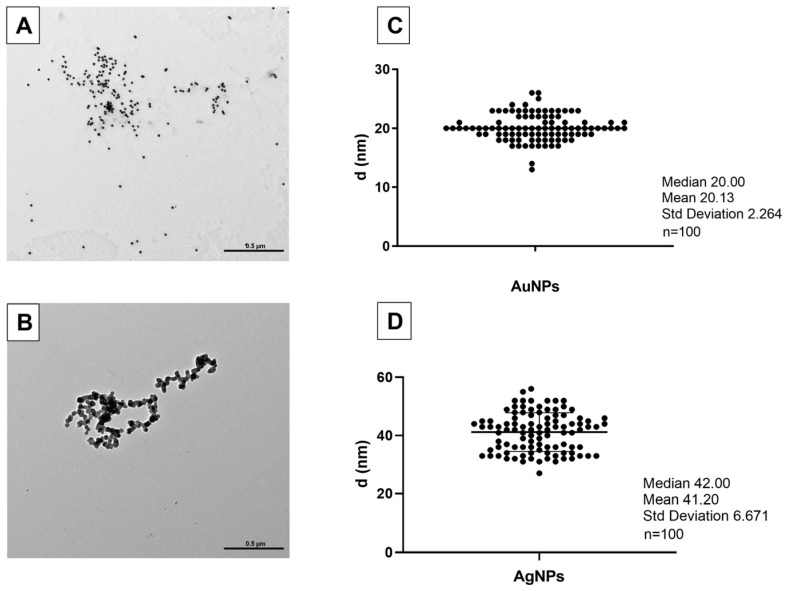
(**A**,**B**) Transmission electron microscopy micrographs of AuNPs and AgNPs diluted in FETAX solution; (**C**,**D**) average mean of nanoparticles diameters (d). One hundred NPs of each type were measured (n = 100). Bars: 0.5 µm.

DLS measurement shows that AuNPs (1 mg/L) have an average diameter of 30 nm in H_2_O and that they form dimers in FETAX solution. These data are confirmed by the z-potential values that ranged at −35.8 in H_2_O and −24.37 in the FETAX solution. Data in the literature report that, at z-potential values of ±30 mV, electrostatic interactions between particles are strong enough for electrostatic stability, while at intermediate values, near their isoelectric point, particles can flocculate [37]. The polydispersity index (PdI) values, greater than 0.2, indicate that the aggregates present in the solution are unevenly distributed (Table 2). However, according to TEM images and measurements, AuNP diameters ranged around 20 nm, as indicated by the manufacturer (Figure 1A,C). No aggregates were visible.

AgNPs observed using TEM showed an average diameter of about 41 nm (Figure 1B,D), contrary to that declared by the manufacturer (20 nm). Aggregates were occasionally observed. In DLS data for the AgNPs (1 mg/L), the z-potential values, measured within 1h, ranged at circa −25.70 and −22.43 mV in H_2_O and FETAX, respectively, values allowing particle aggregation. Indeed, the z-average indicates the presence of aggregates of about 174.8 nm in FETAX and circa 67 nm in H_2_O. Furthermore, the PdI values (0.3840 and 0.4297, respectively) indicate that the aggregates present in the solution are unevenly distributed (PdI > 0.2) (Table 2).

### 3.2. AuNPs and AgNPs Are Not Lethal

**Figure 2:**
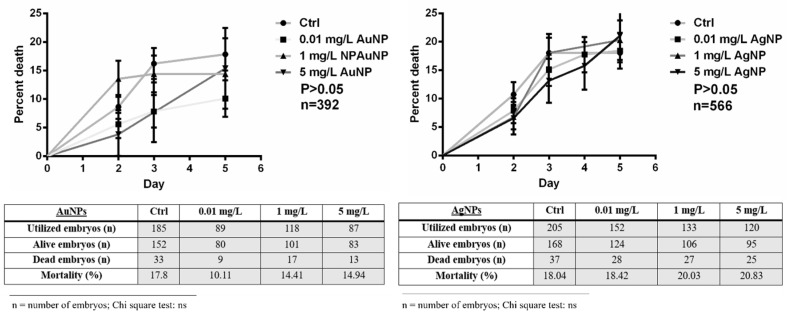
Survival of embryos raised in the presence of AuNPs and AgNPs. The experimental points represent the average of three independent experiments, and the error bars indicate the standard error. No significative differences were reported among control groups and NPs treated. n = total number of embryos analyzed.

Following the monitoring and day-to-day counting of embryos, it emerged that AgNPs and AuNPs do not influence the mortality rate. No statistically significant differences were detected between the control embryos and those treated with AuNPs or AgNPs, for all concentrations (*p* > 0.05; Figure 2).

### 3.3. Exposure to AuNPs and AgNPs Induced Length and Pigment Variation and Heart Rate Alterations

**Figure 3:**
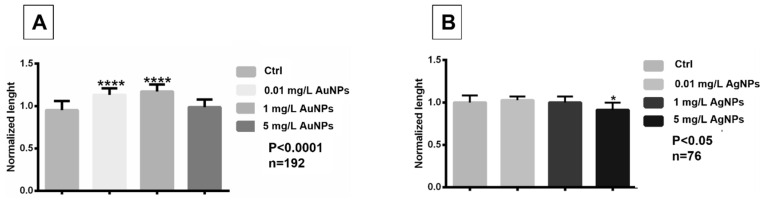
(**A**,**B**) Variation in embryo length collected at stage 45/46 following the exposure to AuNPs and AgNPs. Data are means ± standard deviation of three independent experiments. * *p* < 0.05; **** *p* < 0.0001. n = total number of embryos analyzed.

AuNPs treatment at 0.01 mg/L and 1 mg/L induced a significant increase in embryo length (*p* < 0.0001) while at 5 mg/L, no differences with controls were observed (Figure 3A). In contrast, embryos treated with AgNPs at the two lower concentrations did not show significant differences in length compared to controls, while after treatment with 5 mg/L, they were significantly shorter than the controls (*p* < 0.05) (Figure 3B).

**Figure 4:**
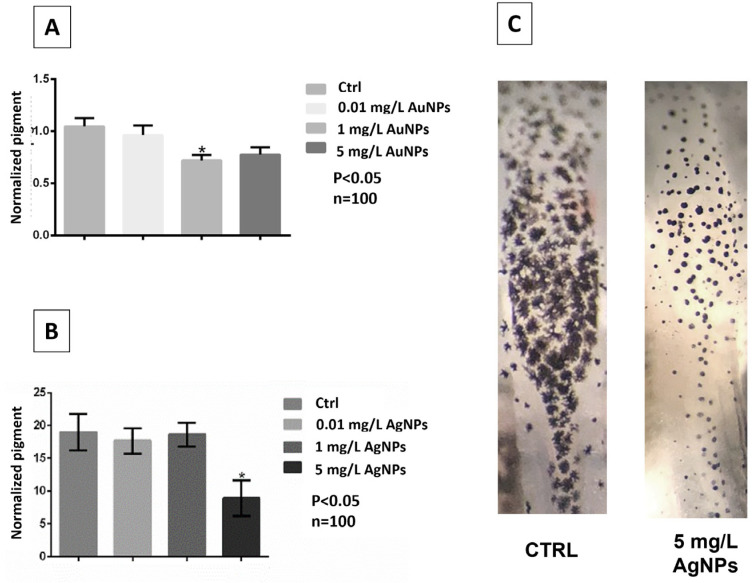
(**A**,**B**) Pigment intensity (mean and standard deviation) in embryos treated with AuNPs or AgNPs, collected at stage 45/46; (**C**) distribution and shape of pigment in the dorsal area in control and 5 mg/L AgNP-treated embryos. Data are means ± standard deviation of three independent experiments. * *p* < 0.05. n = total number of embryos analyzed.

The variation in the dorsal pigment of the embryos treated at different concentrations of AuNPs and AgNPs is reported in Figure 4. In AuNPs-treated embryos, the pigment showed a moderate decrease at the two high concentrations (Figure 4A). For AgNP-treated embryos, a significative change was detected at 5 mg/L concentration, where embryos displayed strong depigmentation (Figure 4B,C). In Figure 4C, the dorsal view of embryos treated with AgNPs showed a change in the number of melanocytes and in the concentration of pigment within them or even the arrangement of the pigment itself.

**Figure 5:**
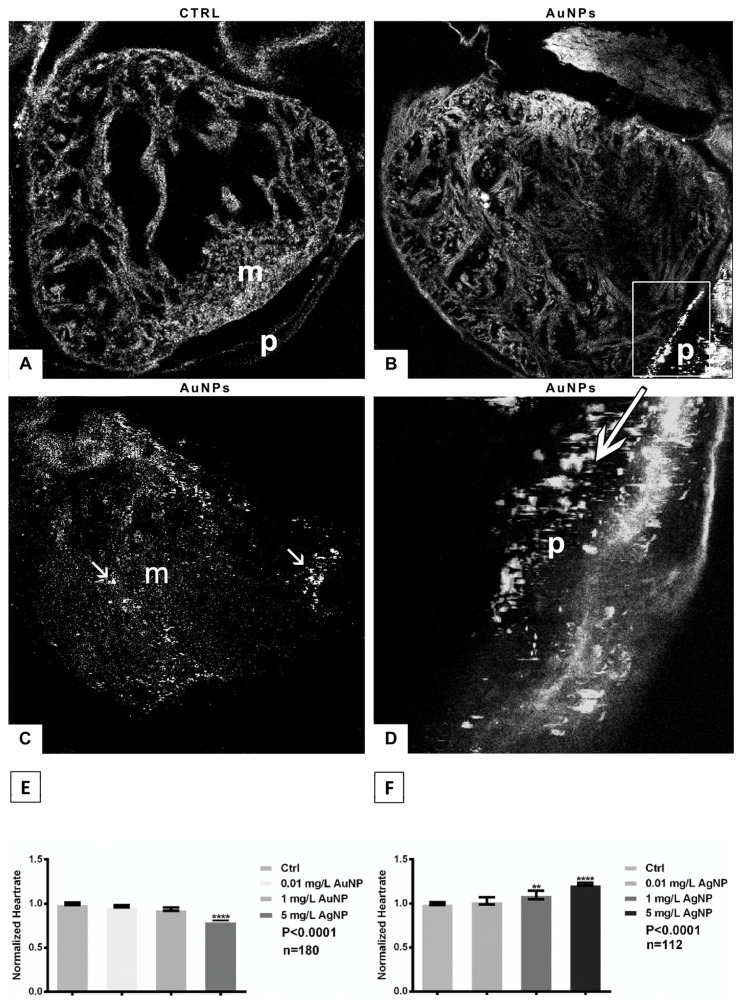
(**A**) Confocal microscopy of the control heart where myocardium (m) and pericardium (p) are visible; (**B**,**C**) in AuNP-treated embryos, nanoparticles penetrate the organ and are detected in both the myocardium and pericardium (arrows); (**D**) vibration of the nanoparticles in the pericardium (arrow); (**E**) significant bradycardia in embryos treated with 5 mg/L AuNPs, collected at stage 45/46; (**F**) significant tachycardia in embryos exposed to the two highest concentrations of AgNPs, collected at stage 45/46. Data are means ± standard deviation of three independent experiments. ** *p* < 0.01; **** *p* < 0.0001. n = total number of embryos analyzed.

Confocal microscopy showed that the AuNPs were distributed in the pericardium (Figure 5B) and in the myocardium (Figure 5C), where magnification (Figure 5D) revealed the vibrations of the nanoparticles, caused by laser stimulation. For heart rate, exposure to 5 mg/L AuNPs induced bradycardia (*p* < 0.0001, Figure 5E), while tachycardia was observed after AgNP exposure, especially for embryos treated with 5 mg/L (*p* < 0.0001, Figure 5F).

### 3.4. AuNPs and AgNPs Caused Intestinal Damage

**Figure 6:**
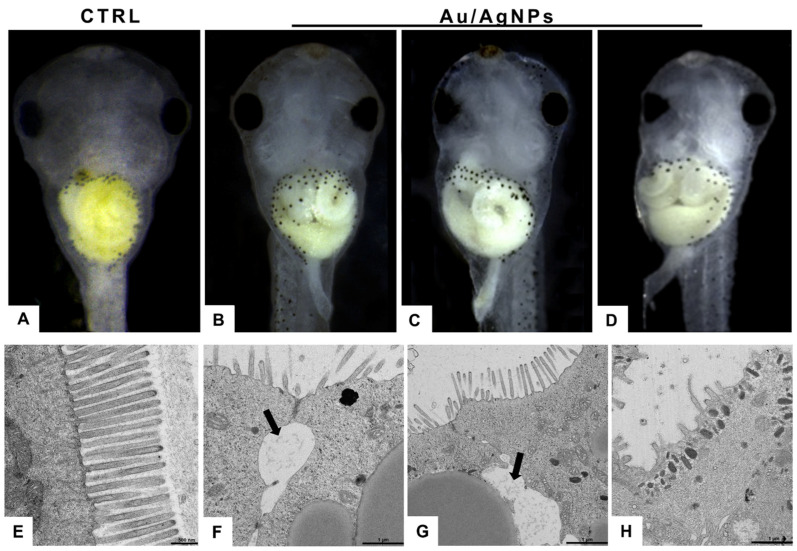
(**A**) Correct folding of the intestine in control embryos at stage 45/46; (**B**–**D**) treatment with AuNPs or AgNPs induces defects in maturation, causing altered morphologies concerning controls; (**E**) TEM micrograph of the control intestine; note the normal organization of microvilli; (**F**–**H**) in embryos exposed to both AuNPs or AgNPs, the microvilli are altered, and enterocytes have partly lost connection with the consequent appearance of large intercellular areas (arrows).

Normally, embryos at stage 46/46 have a convoluted intestine, with 2–2½ revolutions (Figure 6A). In treated embryos, no matter the type of NPs, the intestine appeared poorly or abnormally winded (Figure 6B–D). Transmission electron microscopy revealed, in controls, the presence of a brush border with long microvilli (Figure 6E). In treated embryos, shorter or completely missing microvilli indicated signs of suffering, compatible with mechanical damage exerted by the nanoparticles (Figure 6F–H). The enterocytes, in some cases, lost contact, in particular in the apical region, determining the appearance of large areas with clear infiltrates (Figure 6F).

### 3.5. AuNPs and AgNPs Modified Skull–Facial Cartilages and Branchial Basket Conformation

**Figure 7:**
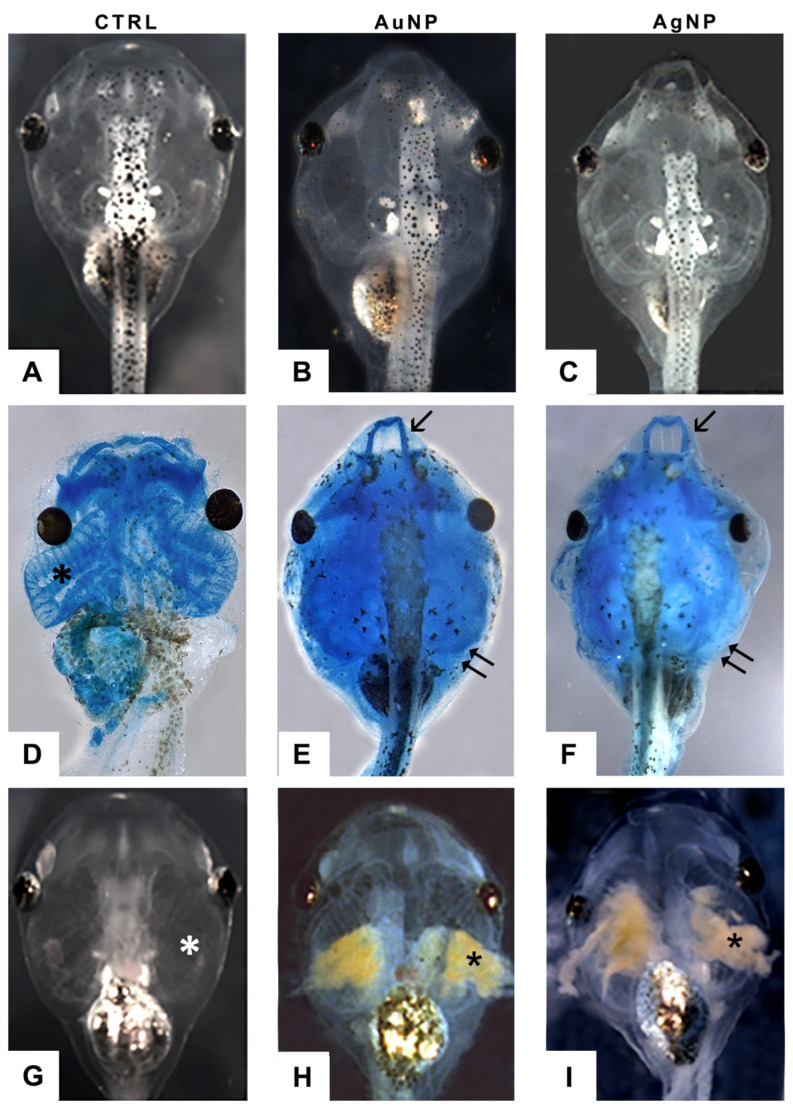
(**A**) Dorsal view of a control embryo and embryos treated with (**B**) AuNPs or (**C**) AgNPs at stage 45/46; (**D**) dorsal view, control embryo; well-developed gill basket (*); (**E**,**F**) dorsal view, AuNP- and AgNP-treated embryos with deformed Meckel’s cartilage (arrows) and gill basket (double arrows); (**G**) ventral view, control embryo; gills (*) are transparent; (**H**,**I**) ventral view, treated embryos; gills are obstructed, respectively, by AuNPs and AgNPs (*). (**D**–**F**): Alcian Blue staining.

Alcian Blue staining highlighted the occurrence of malformations in the facial and gill basket cartilages (Figure 7). After treatments with both NP types, the skull was compressed laterally, and the Meckel’s cartilage (mandible) protruded from the head contour (Figure 7E,F). The cartilaginous gill basket was expanded in embryos treated with AuNPs (Figure 7E) or flattened in embryos treated with AgNPs (Figure 7F) or even not perfectly visible, probably due to the accumulation of stained NPs at the level of the gills. Indeed, after both treatments, it was possible to notice a strong opaque coloring (Figure 7H,I), not present in controls (Figure 7G), due to the concentration of NPs in these filtering organs. It is also possible to notice a reduction in the size of the eye, especially in the treatment with silver nanoparticles (Figure 7F).

### 3.6. No Oxidative Stress Is Induced by AuNPs and AgNPs Exposure

**Figure 8:**
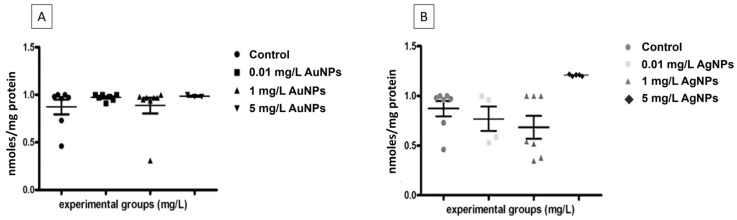
Reactive oxygen species in embryos exposed to AuNPs (**A**) or AgNPs (**B**) expressed as nanomoles of TBARS per milligram of protein. No significant increase in ROS levels was found compared to the controls. Data are means ± standard deviation of three independent experiments and were collected at stage 45/46.

To determine if NPs induced oxidative stress, the TBARS quantization assay was performed. The analysis showed that the embryos exposed to AuNPs (Figure 8A) or AgNPs (Figure 8B) did not change ROS levels; the only exceptions were the embryos treated with 5 mg/L of AgNPs that showed a small but not significative production of malondialdehyde (MDA) (Figure 8B).

### 3.7. Exposure to AuNPs and AgNPs Altered Gene Expression

**Figure 9:**
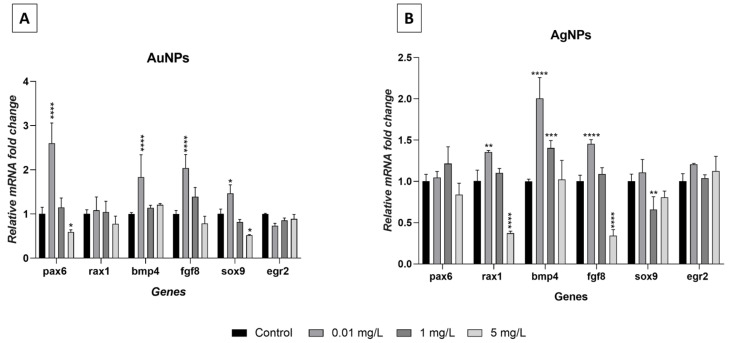
Gene expression analysis in *Xenopus laevis* embryos (stage 45/46) exposed to AuNPs (**A**) or AgNPs (**B**). Results are the means ± SEM of three independent experiments. * *p* < 0.05; ** *p* < 0.01; *** *p* < 0.001; **** *p* < 0.0001.

The phenotypic modifications observed in the embryos treated with AgNPs or AuNPs involve derivatives of the ectoderm, mesoderm, and endoderm. For this reason, attention has been paid to the expression of a group of master genes involved in early embryonic development (Figure 9). Results indicated that exposure to NPs determined significant variations in the expression of almost all the genes analyzed. Treatment with AuNPs (Figure 9A), at a concentration of 0.01 mg/L, induced a significant overexpression of *pax6*, *bmp4*, *fgf8* (*p* < 0.0001), and *sox9* (*p* < 0.05). At the same concentration, the embryos treated with AgNPs (Figure 9B) displayed overexpression of *rax1* (*p* < 0.001), *bmp4*, and *fgf8* (*p* < 0.0001). At the intermediate concentration of 1 mg/L, AgNPs altered the expression of *bmp4* and *sox9* that were overexpressed (*p* < 0.001) and downregulated (*p* < 0.01), respectively. At the highest concentration of 5 mg/L, hypoexpression was observed for *pax6* and *sox9* (*p* < 0.05) after AuNP exposure and for *rax1* and *fgf8* (*p* < 0.0001) after AgNP exposure. For both treatments, *egr* always displayed normal expression levels.

## 4. Discussion

In recent decades, nanotechnologies have aroused increasing interest from the scientific community for the revolutionary perspectives/applications related to their use. The extremely small dimensions, in fact, drastically change the characteristics of the different materials, allowing applications so far unpredictable. On the other hand, it is now clear that their indiscriminate use could represent a health risk since they are not biodegradable, can bioaccumulate, and produce damage [38,39]. In our study, we verified the negative effects of AuNPs and AgNPs on *Xenopus laevis* embryo development.

The data obtained from DLS and TEM are contradictory. The evidence collected using DLS shows that both AuNPs and AgNPs, suspended in H_2_O or FETAX medium, have diameters larger than those stated by the manufacturer (20 nm). In contrast, in the case of AuNPs, TEM investigations indicate a size identical to that declared by the manufacturer. Another discrepancy evidenced by the two techniques is the tendency to form aggregates. DSL indicates that both NPs form aggregates, nonhomogeneous in size, while in TEM, aggregates were never observed. At the moment, is difficult to find an explanation for these differences. DLS may read the hydration shell as part of the NPs and then show a major diameter of nanoparticles [40], while for TEM investigations, the spreading of NPs on grids may mechanically disperse aggregated particles. Contrasting results confirm that the current analytical techniques for detecting NPs still need to improve in reliability and accuracy [41]. Of particular interest is the evidence that NPs behave differently in water and saline FETAX medium [42]. Particle size and aggregation are of great relevance since these control penetration and distribution in the organism: if small, particles are rapidly cleared by excretory mechanisms while if large, they are eliminated by phagocytosis [43] with longer residence times in organisms. We must consider that toxicity is linked to the time of residence in the organism [44], as is the ability to transfer into the tissues the toxicants present in the environment [45].

Data on *Xenopus* development indicate that NPs have no effects on mortality rate. Other authors have reported that AuNPs are very biocompatible, nontoxic, and even able to improve health status by increasing, for example, antioxidant capacity and immunity [46,47]. The reduced/lack of toxicity for AuNPs would depend on surface charges [41]. In *Xenopus* [48], positively charged Au-PEI25kB exhibited significant toxicity and teratogenicity, whereas uncharged AuNPs did not. In our experiments, a moderate, not significant decrease in mortality was registered in treated larvae concerning controls. A different situation was found for AgNPs. Our data on *Xenopus* is not in line with the literature reporting high toxicity for these NPs: in zebrafish, they induce 50% mortality at 3 mg/L [49] and significant toxicity has also been reported in rats [50]. In *Xenopus* embryos, mortality does not increase significantly; however, it did increase with respect to controls. This evidence suggests that, in this case, toxicity is related to dosage.

Another factor to be considered is the concentration. In *Xenopus* embryos, positively charged BPEI-AgNPs induce malformations in a concentration-dependent manner [51]. In our experiments, data on growth indicate that AuNPs have a positive influence at low concentrations, probably in relation to the positive effects they exert on oxidative stress, immunity [47], and stress response [52]. The absence of effects at the higher concentration indicates a hormesis effect [53]: AuNPs positively influence the embryos if administered at a low dose and stimulate metabolic pathways without inducing toxicity. In contrast, no such positive effects were registered for AgNPs that inhibited growth at the higher dose. The opposite effects of Au and AgNPs can be related to the different interference in cell metabolism and energy homeostasis, in anabolic or catabolic processes [54].

*Xenopus* embryos internalized both AuNPs and AgNPs when they were still enclosed in the fertilization envelope; the entrance site would be the skin and gills [4,49,55,56]. NPs diffused in the entire organism (were found in the heart) and exerted marked toxicity on the gut that resulted in poor/irregular coiling. Damage was accompanied by parallel modification of *bmp4* expression, mainly responsible for gut architecture [57], via *foxf1* [58,59].

NPs also entered by direct ingestion, after stage 41, when the stomodeum opened [58]. By encountering enterocytes, they damage microvilli, thus reducing functionality and altering energy metabolism [60]. Further proof of embryonic distress is evidenced by the altered heart rate [61]. Alterations accord with the presence of NPs in the myocardium and with the altered expression of *fgf8*, *bmp4*, and *sox9*, genes required for the proper development of the cardiovascular system [62,63,64]. From confocal microscopy, no structural damage was observed but further investigations are necessary, using the light and electron microscope, to verify that this is the case. Beat alteration is accompanied by altered anatomy of the branchial region and accumulation of NPs at this level. At the moment, it is not possible to establish whether it is the structural alteration that blocks the NPs or vice versa. In any case, it is easy to hypothesize that the gills have reduced functionality and that gas exchange is altered. Significantly, gaseous imbalance did not lead to oxidative stress, in line with previous reports for gold [65] but not silver [66] nanoparticles. In this case, also it may be a matter of doses, since reactive oxygen species started to increase at the higher concentration tested.

Another evidence of interference of NPs on larval development is altered pigmentation. Melanocyte count did not decrease but melanin appeared aggregated [67,68]. This suggests either some form of neurodegeneration/interference [68] or release of cortisol (AuNPs [52]; AgNPs [69]) or interference in metallothioneins (MTs) or MT transporter activity [70,71]. Changes in larval pigmentation are a toxic response common in *Xenopus* and already observed, for example, after treatment with pesticides [33] or SiO_2_NPs [4] or exposure to UV [72]. Depigmentation observed after AuNP and AgNP exposure is particularly relevant, since it would reduce camouflage and, in the wild, could be detrimental to survival.

Analyses confirm that AuNPs and AgNPs induce significant variations in the expression of developmental master genes, thus accounting for the observed structural alterations in the gut and skull. The cartilaginous lower jaw and gill basket are profoundly altered, suggesting a specific effect of NPs on the development of branchial arches I and II and, in particular, on the expression of genes involved in the neural crest’s differentiation and movement during neurulation [73,74]. Currently, neural crests are considered a true embryonic leaflet because of their unique characteristic of giving rise to tissues with different fates [24]. In exposed embryos, these structures were correctly located, suggesting that the neural crests cells migrated correctly, as also indicated by the unmodified expression of *egr2.* The observed defects would rather depend on altered expression of *fgf8*, *bmp4*, and *sox9*, genes that are essential for the outgrowth of maxillary and frontonasal skeletal elements [75,76,77].

The observed alterations in *pax6* expression correlate well with the observation that larvae have eyes of reduced size. This structure is thus confirmed as a specific target for xenobiotics of various natures [22,25,78]. In contrast, altered expressions of *bmp4* and *fgf8* did not determine any structural alteration in body patterning though *fgf/bmp* and *wnt* pathways regulate the patterning of embryo axes [79,80,81].

## 5. Conclusions

In conclusion, the experimental data collected demonstrate that AuNPs and AgNPs, at the doses tested, are not lethal to embryos but profoundly alter development, as indicated by the abnormal phenotypes. Altered pigment distribution, malformations at the level of facial cartilage and gill basket, immaturity of the intestine, and changes in heart rate all depend on the altered expression of master developmental genes responsible for the ‘construction’ of the tadpole. Data in the literature show that increased ROS production is one of the causes of toxicity for NPs, but this was not the case here. Further studies are therefore required to fully understand NP behavior in nature and in tissues and to comprehend the effects they exert on amphibians in particular and aquatic organisms in general.

## Figures and Tables

**Table 1:** Primers used to perform real-time PCR.

Gene Name	Oligo Forward Sequence	Oligo Reverse Sequence
*bmp4*	CCTCAGCAGCATTCCAGAGAA	TCCGGTGGAAACCCTCATCC
*fgf8*	CGTTTGGAAGCAGAGTTCGC	GTTGCCTTGTCTTCGACCCT
*sox9*	ACGGCGCAGAAAGTCTGTTA	GACATCTGTCTTGGGGGTGG
*egr2*	AGTAAGACCCCAGTCCACGA	GCAGTAATCGCAGGCAAAGG
*pax6*	CAGAACATCTTTTACCCAGGA	GAATGTGGCTGGGTGTGTTA
*rax1*	GGAAAGACCTCAAGCGAGTG	ATACCTGCACCCTGACCTCG
*odc1*	GTGGCAAGGAATCACCCGAA	TCAAAGACACATCGTGCATC

**Table 2:** DLS of AuNPs and AgNPs in H_2_O and FETAX solution.

AuNPs	Size [nm]	Size [SD]	Z-Potential [mV]	Z-Potential [SD]	PdI	PdI [SD]
H_2_O	32.76 ± 4.0.96 ^a^	1.67	−35.8 ± 1.36°	2.35	0.35 ± 0.024°	0.04
FETAX	62.08 ± 0.35 ^a^	0.60	−24.37 ± 1.43°	2.48	0.48 ± 0.003°	0.006
						
**AgNPs**	**Size** **[nm]**	**Size** **[SD]**	**Z-Potential [mV]**	**Z-Potential [SD]**	**PdI**	**PdI** **[SD]**
H_2_O	66.98 ± 4.198 ^a^	7.271	−25.70 ± 3.256°	5.640	0.3840 ± 0.04596°	0.07961
FETAX	174.8 ± 42.23 ^a^	73.14	−22.43 ± 0.9244°	1.601	0.4297 ± 0.07917°	0.1371

^a^ Mean value ± SE; n = 3. SD = standard deviation.

## Data Availability

The data are contained within the article.
